# Characterization of a novel glycosylated glutathione transferase of *Onchocerca ochengi*, closest relative of the human river blindness parasite

**DOI:** 10.1017/S0031182019000763

**Published:** 2019-07-03

**Authors:** Clair Rose, Giorgio Praulins, Stuart D. Armstrong, Aitor Casas-Sanchez, Jem Davis, Gemma Molyneux, Cristina Yunta, Zenaida Stead, Mark Prescott, Samirah Perally, Anne Rutter, Benjamin L. Makepeace, E. James La Course, Alvaro Acosta-Serrano

**Affiliations:** 1 Liverpool School of Tropical Medicine, Liverpool, UK; 2 Institute of Infection & Global Health, University of Liverpool, Liverpool, UK; 3 Institute of Integrative Biology, University of Liverpool, Liverpool, UK

**Keywords:** Detoxification, glycans, glycosylation, GSTs, immune modulation, *Onchocerca*, prostaglandin synthase

## Abstract

Filarial nematodes possess glutathione transferases (GSTs), ubiquitous enzymes with the potential to detoxify xenobiotic and endogenous substrates, and modulate the host immune system, which may aid worm infection establishment, maintenance and survival in the host. Here we have identified and characterized a *σ* class glycosylated GST (OoGST1), from the cattle-infective filarial nematode *Onchocerca ochengi,* which is homologous (99% amino acid identity) with an immunodominant GST and potential vaccine candidate from the human parasite, *O. volvulus*, (OvGST1b). *Onchocerca ochengi* native GSTs were purified using a two-step affinity chromatography approach, resolved by 2D and 1D SDS-PAGE and subjected to enzymic deglycosylation revealing the existence of at least four glycoforms. A combination of lectin-blotting and mass spectrometry (MS) analyses of the released *N*-glycans indicated that OoGST1 contained mainly oligomannose Man_5_GlcNAc_2_ structure, but also hybrid- and larger oligommanose-type glycans in a lower proportion. Furthermore, purified OoGST1 showed prostaglandin synthase activity as confirmed by Liquid Chromatography (LC)/MS following a coupled-enzyme assay. This is only the second reported and characterized glycosylated GST and our study highlights its potential role in host-parasite interactions and use in the study of human onchocerciasis.

## Introduction

The cattle filarial nematode *Onchocerca ochengi* is a well-established model natural system for the study of human onchocerciasis, the causative agent of which is *O. volvulus* (Trees, 1992; Makepeace and Tanya, 2016). Onchocerciasis is a devastating, vector borne, neglected tropical disease, affecting over 15 million people, 99% of whom live in Africa. Symptoms range from severe itching and disfiguring skin conditions (for the majority of sufferers), to being the second-leading infectious cause of blindness in Africa at over 1 million afflicted with vision loss (WHO, 2018). In efforts to discover new ways to control onchocerciasis, much research has been focused on the molecules which may allow *Onchocerca* spp. to establish and maintain infection. One such protein family is the glutathione transferases (GSTs) which may aid worm survival through detoxification of drugs and evasion of host-derived immunochemical attack, and with the potential to play roles in immunomodulation (Chasseaud, 1979; Jakoby and Habig, 1980; Brophy and Barrett, 1990; Sheehan *et al*., 2001; Sommer *et al*., 2003; Hayes *et al*., 2005). Initial explorations of the *Onchocerca* spp genomes reveal a glutathione transferase (OoGST1 – Accession, nOo.2.0.1.t09064) from *O. ochengi* (Armstrong *et al*., 2016), displaying 99% amino acid identity with an immunodominant GST (OvGST1b – Accession AAG44696.1) (Liebau *et al*., 1994; Alhassan *et al*., 2014) and potential vaccine candidate from the closely related human parasite *O. volvulus* (Graham *et al*., 1999). OvGST1b and its paralogous gene product OvGST1a (AAG44695.1) are exceptional within the GST superfamily in being glycosylated, having a cleavable signal peptide and N-terminal extension not found in other GSTs, though the roles of these novel features and glycosylation are yet to be fully elucidated (Sommer *et al*., 2003; Perbandt *et al*., 2008). The potential of these inherent glycans to function in, as yet undefined, roles at the host-parasite interface are of particular interest given OvGST1b is shown to have prostaglandin synthase activity and so may possess the ability to modulate the host immune response to filarial infection (Sommer *et al*., 2003; Perbandt *et al*., 2008). Furthermore, a deeper knowledge of protein glycosylation has important implications for future vaccine development and an understanding of host-parasite interactions. Whilst most screening of parasite products for immunoreactive vaccine candidate antigens is predominantly protein focused (Diemert *et al*., 2018), antibody responses to glycosylated proteins demonstrates the high immunogenicity of glycan extensions, highlighting the clear rationale for a greater attention (Jaurigue and Seeberger, 2017).

Extending such focus on OvGST1b in the human-infecting *O. volvulus* filarial worm is however significantly limited through obvious logistical aspects in access to worm samples and necessary ethical constraints. Many onchocerciasis studies thus employ the closely related *O. ochengi* species in cattle as a valuable and accessible model for research into the genes and proteins which may play roles in this disease (Trees *et al*., 2000). *Onchocerca ochengi* GST homologue OoGST1 however, has yet to be isolated and studied to validate its status in terms of comparable structure, glycosylation state and enzymic activity with that of OvGST1b.

Therefore, investigations to resolve *O. ochengi* GSTs, characterize the enzymic activity and unravel the structure of *N*-glycan modifications of the homologous OoGST1 protein, are presented here to allow comparative exploration of potential roles in host-parasite interactions. Our findings reveal subtle differences in glycosylation state between OvGST1s and OoGST1.

## Materials and methods

### Parasite material

*Onchocerca ochengi* adult female gravid worms were collected from nodules in hides of infected Gudali cattle from the Ngaoundéré abattoir in the Adamawa region of Cameroon. Worm masses were dissected from collagenous tissue within nodules and male worms were removed. The females were washed in PBS and separated into 2 mL cryovial tubes before freezing and storage at −80 °C. Isolated female worms were transported to the UK on dry ice.

### Glutathione transferase purification

Cytosolic extracts from *O. ochengi* were obtained by homogenization of frozen worms in an ice-cooled glass grinder in buffer containing 20 mm potassium phosphate, pH 7.0, 0.2% Triton X-100, 5 mm DTT and a cocktail of protease inhibitors (Roche, Mini-Complete, EDTA-free). Following homogenization, samples were centrifuged at 100 000 × ***g*** for 1 h at 4 °C and the supernatant, termed the cytosolic fraction, was retained for purification of GSTs.

GSTs were partially purified and further resolved to isolate glycosylated forms from the cytosolic fraction in two steps; ‘*Step 1*’ employed *S*-hexylglutathione-affinity (*S*-hexylGSH-affinity) chromatography according to the adapted method of (Simons and Vander Jagt, 1977). In brief, the *O. ochengi* cytosolic fraction was passed at 0.5 mL min^−1^ through Econo-columns (1.0 × 5 cm, 4 mL Bio-Rad, U.K.), containing 1 mL of *S*-hexylGSH–agarose (Sigma Aldrich), re-hydrated according to manufacturer's instructions and equilibrated with 20 mL of 20 mm potassium-phosphate buffer pH 7.0, 50 mm NaCl (equilibration buffer). Non-*S*-hexylGSH-affinity proteins were washed from the column with 20 mL equilibration buffer at 0.5 mL min^−1^. Affinity-bound proteins were eluted in 3 mL 50 mm Tris-HCl pH 8.0 buffer, containing 2 mm *S*-hexylGSH, and concentrated *via* centrifugal filtration in 10 kDa molecular weight cut off filters (Amicon Ultra-4, Millipore). GST samples were reduced to a final volume of 100 *µ*L through three successive cycles of ten-fold dilutions/centrifugal reductions in 50 mm Tris-HCl pH 8.0 to remove proteins, free glutathione and low molecular mass substances of a native weight below 10 kDa.

The partially purified pool of GSTs obtained in ‘*Step 1’* was incubated with a range of different lectins to determine optimum lectin selection for ‘*Step 2*’ isolation of glycosylated GST from the GST pool. Glycosylated GSTs were isolated from the partially purified pool of GSTs obtained in ‘*Step 1*’ *via* lectin-affinity chromatography using concanavalin A-agarose (Sigma Aldrich) according to the manufacturer's instructions.

### Glutathione transferase enzyme activity

Establishment of GST presence within *O. ochengi* cytosolic extracts and S-hexylglutathione-binding protein samples was assayed *via* enzyme activity at 25 °C over 3 min at 340 nm using 1 mm 1-chloro-2, 4-dinitrobenzene (CDNB) as standard second substrate in 100 mm potassium phosphate pH 6.5, containing 1 mm reduced glutathione in accordance with the adapted method of Habig *et al*. (1974). Assays were undertaken in triplicate in a Cary Varian spectrophotometer with specific activity expressed as nmol GSH/CDNB conjugated min^-1^ mg^-1^ protein (± standard deviation), and calculated as described by (Barrett, 1997) (see equation (1) below):1
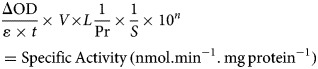
*Key to equation (1).* ΔOD = change in optical density over time (*t*) in min; *ε* = extinction coefficient; *V* = total volume of assay mixture in the cuvette (mL); *L* = path length of the cuvette in cm; Pr = protein concentration of enzyme extract (mg mL^−1^); *S* = volume of enzyme extract added to a cuvette (mL); The value of *n* is dependent on the extinction coefficient (*ε*): If *ε* is in cm^2^ M^−1^, then *n* = 9, If *ε* is in M^−1^ cm^−1^, then *n* = 6, If *ε* is in mm^−1^ cm^−1^, then *n* = 3)

Protein concentrations were estimated *via* the adapted method of Bradford (Bradford, 1976) using the Sigma (UK) Bradford Reagent protocol according to the manufacturer's instructions.

### Electrophoresis

#### Two-dimensional gel electrophoresis (2DE)

20 *µ*g of native purified GSTs (S-hexylglutathione-binding proteins) was resuspended into immobilized pH gradient (IPG) rehydration buffer [6 M urea, 1.5 M thiourea, 3% w/v CHAPS, 66 mm DTT, 0.5% v/v ampholytes pH 3–10 (Pharmalytes, Amersham BioSciences, UK)] to a final volume of 300 *µ*L. In-gel passive rehydration and isoelectric focusing of IPG gel strips with protein samples was at 20 °C with mineral oil overlay according to IPG strip manufacturer's instructions (Bio-Rad, UK). Isoelectric focused strips were equilibrated, in two stages: a ‘reducing stage' for 15 min in ‘equilibration buffer’ (50 mm Tris–HCl pH 8.8, 6 M urea, 30% glycerol, 2% SDS) containing 1% (w/v) DTT, followed by a 15 min ‘alkylating stage’ in ‘equilibration buffer’ containing 2.5% (w/v) iodoacetamide replacing 1% DTT (LaCourse *et al*., 2009). Gels were then fixed overnight in 40% methanol/10% acetic acid, stained in colloidal Coomassie Blue G-250 overnight and then de-stained in 1% acetic acid.

#### Sodium dodecyl sulphate polyacrylamide gel electrophoresis (SDS-PAGE)

Protein samples were resolved by SDS-PAGE according to methods adapted from Laemmli (1970) on 12.5% polyacrylamide gels as previously described (LaCourse *et al*., 2009). Gels were Coomassie or Periodic-acid Schiff (PAS) stained (Sigma) and scanned upon a GS-800 densitometer (Bio-Rad).

### Quadrupole time of flight (QToF) tandem mass spectrometry (MS/MS) analysis of OoGST peptides

Tryptic peptides were generated as previously described (LaCourse *et al*., 2009). Peptide mixtures from trypsin digested gel spots were separated using an LC Packings Ultimate nano-HPLC System. Sample injection was *via* an LC Packings Famos auto-sampler and the loading solvent was 0.1% formic acid. The pre-column used was an LC Packings C18 PepMap 100, 5 mm, 100A and the nano HPLC column was an LC Packings PepMap C18, 3 mm, 100A. The solvent system was: solvent A 2% ACN with 0.1% formic acid, and solvent B, 80% ACN with 0.1% formic acid. The LC flow rate was 0.2 mL min^−1^. The gradient employed was 5% solvent A to 100% solvent B in 1 h. The HPLC eluent was sprayed into the nano-ES source of a Waters Q-TOF*μ* MS *via* a New Objective Pico-Tip emitter. The MS was operated in the positive ion ES mode and multiple charged ions were detected using a data-directed MS-MS experiment. Collision induced dissociation (CID) MS-MS mass spectra were recorded over the mass range *m/z* 80–1400 Da with scan time 1 s. The raw MS-MS spectral data files were processed using Waters ProteinLynx software (Waters, UK) to produce Sequest dta file lists which were then merged into a Mascot generic format (mgf) file.

### Protein identification

All tandem MS data generated were searched against partially revised *Onchocerca ochengi* gene models based on data downloaded from WormBase ParaSite (Armstrong *et al*., 2016) and a *Bos taurus* reference proteome (UniProt UP000009136, March 2019) (37957 sequences, 17775113 residues in total) using the search engine MASCOT (version 2.3.02, Matrix science) Search parameters were a precursor mass tolerance of ±1.2 Da and fragment mass tolerance of ±0.6 Da. One missed cleavage was permitted, carbamidomethylation was set as a fixed modification and oxidation (M) and deamidation (N, Q) were included as variable modifications. Individual ion scores ⩾39 were considered to indicate identity or extensive homology (*P* < 0.05), using MudPIT scoring. Only proteins with >2 peptides were used for analysis. Data were deposited to the PRIDE repository (Vizcaino *et al*., 2016) with the data set identifier PXD013440.

### Glycan analysis of *O. ochengi σ* class GST

#### Enzymic de-glycosylation of GSTs

GST samples were de-glycosylated with either peptide N^4^-(N-acetyl-*β*-glucosaminyl) asparagine amidase *F* (PNGase *F*) or endoglycosidase *H* (Endo *H*) (both from NEB) treatment under reducing conditions according to the manufacturer's instructions. Briefly, 0.4 *µ*g *µ*L^−1^ OoGST and 1 *µ*g *µ*L^−1^ of glycosylated egg albumin (as positive control) were denatured at 100 °C for 10 min in Glycoprotein Denaturing Buffer. NP-40 was then added for PNGase F treatment only and samples digested with 25 units *µ*L^−1^ per enzyme overnight in a 37 °C water bath. Mock-treated samples were processed the same (but without the addition of any enzyme) and the reactions were stopped by heating. All protein samples were fractionated by SDS-PAGE and either Coomassie blue or PAS stained or used for lectin blotting as indicated below.

#### Lectin-blotting

Lectin blotting was performed according to methods adapted from (Luk *et al*., 2008). Approximately 1 *µ*g of PNGase F-treated or untreated *σ* class OoGST (see above) was fractionated on a 12.5% SDS-PAGE gel as described previously and transferred onto polyvinylidene fluoride (PVDF) membranes at 90 V for 30 minutes on ice. The membranes were then incubated overnight at 4 °C in blocking buffer (PBS, 0.1% (v/v) Tween 20, 1% (w/v) BSA). Following several washes in washing buffer (PBS/0.1% (w/v) Tween 20), each membrane was incubated with 1 *µ*g mL^−1^ biotinylated concanavalin A (ConA) (Vector Labs) for 1 h at room temperature (20–23 °C). Following further washes, membranes were then incubated in streptavidin-horse radish peroxidase (HRP) (ThermoFisher) at a 1: 100 000 dilution for 1 hour at room temperature (20–23 °C). Membranes were washed and then incubated with SuperSignal West Dura (Pierce, UK) peroxidase buffer and luminol:enhancer solution at a 1:1 ratio, and developed by chemiluminescence, which continued for up to 5 h.

### Glycan structural analysis

*N*-glycans from OoGST were released by PNGase F and purified by gel filtration chromatography as indicated in Kozak *et al*., 2015. Hydrophilic Interaction Liquid Chromatography – Ultra high-performance liquid chromatography (HILIC-UHPLC) analysis was performed using a Dionex Ultimate 3000 UHPLC instrument. The conditions included using a BEH-Glycan 1.7 32 *µ*m and 2.1 × 150 mm column at 40 °C, with a fluorescence detector (lamdaex = 310 nm and lamdaem = 370 nm). These conditions were controlled by Bruker HyStar 3.2 buffer A (50 mm ammonium formate pH 4.4) and Buffer B (acetonitrile). Sample volume for injection was 25 *µ*L^−1^, at a ratio of 24 and 76% acetonitrile. Glucose unit (GU) values of peaks were assigned by chromeleon 7.2 data software with a cubic spline fit. The system standard and the GU calibration standard was a glucose homopolymer labelled with procainamide. The mass spectra were collected in a Bruker AmaZon Speed ETD electrospray mass spectrometer, performed immediately after the UHPLC fluorescence detector without splitting. Samples were scanned in maximum resolution mode, positive ion settings, MS scan + three MS/MS scans. The MS/MS scans were done on three ions in each scan sweep with a mixing time of 40 ms at a nebulizer pressure of 14.5 psi, a nitrogen flow of 10 litres min^−1^ and using 4500 V capillary voltage.

### Prostaglandin-synthase assay

Prostaglandin synthase activity was assessed *via* the composite method of LaCourse *et al*. (2012) based upon aspects adapted from the original methods of Sommer *et al*. (2003), Meyer and Thomas (1995) and Meyer *et al*. (1996), with extraction modifications based upon Schmidt *et al*. (2005).

### Sequence analysis of *O. ochengi* glycosylated glutathione transferase OoGST1

*Onchocerca ochengi* glycosylated *σ*-class GST (OoGST1) amino acid sequence (accession number nOo.2.0.1.t09064) was obtained from the University of Edinburgh's *O. ochengi* genome assembly *v*. nOo.2.0.1 hosted by WormBase ParaSite (Howe *et al*., 2017; Consortium, 2019). *Onchocerca ochengi* GST Oo_GST_t09064 was aligned to highlight key residues involved in prostaglandin H_2_ binding using ClustalX Version 2.1 (Thompson *et al*., 1997; Larkin *et al*., 2007) with homologues Ov_GST_Ia (AAG44695.1) and Ov_GST_Ib (AAG44696.1) from *O. volvulus* along with the two mammalian haematopoietic prostaglandin D synthases (PGDS), highlighted in Perbandt *et al*., 2008.

### Structural analysis of OoGST1

Initial protein tertiary models of *O. ochengi σ* class GST were produced *in silico* using SwissModel (Arnold *et al*., 2006) and Phyre2 (Kelley *et al*., 2015) with prostaglandin D synthase of *O. volvulus* (Protein Data Bank (PBD) 2HNL) used as a template structure. Homology prediction was also carried out by RaptorX (Kallberg *et al*., 2012) to predict disordered amino acid regions and I-Tasser (Yang *et al*., 2015) for increased confidence before refinement of the predicted model *via* ModRefiner (Xu and Zhang, 2011). Final structures were modified in PyMol (DeLano, 2002).

## Results

### Sequence analyses and homology prediction

**Fig. 1.**
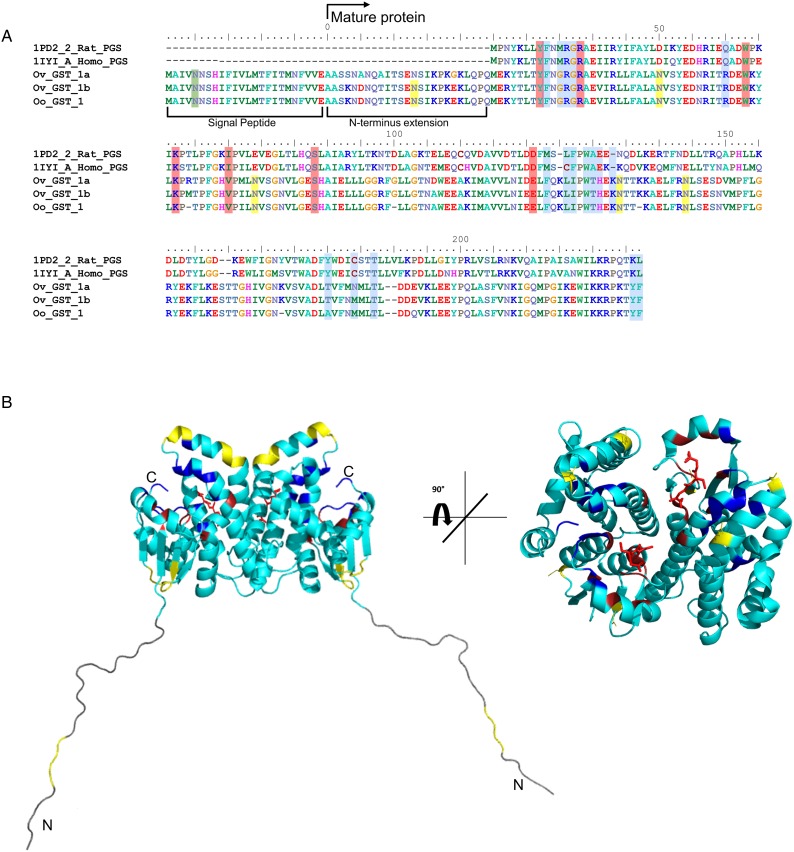
*In silico* analyses of *O. ochengi σ* class GST OoGST1. (A) alignment of amino acid sequences of OoGST1 with homologues from its sister species *O. volvulus*, and PGDS from rat and human. Blue boxes show the regions that are predicted to form the PDH_2_ binding pocket across rat, human and *O. volvulus σ* class GST [information adapted from (Perbandt *et al*., 2008)]. Yellow boxes highlight the predicted *N*-glycosylation sites in the mature *Onchocerca* spp. GSTs, whilst green box highlights a predicted *N*-glycosylation site in the cleavable signal peptide. Red boxes indicate the GSH binding regions. Global alignment was produced using ClustalX Version 2.1 (Thompson *et al*., 1997; Larkin *et al*., 2007). Accession numbers for the proteins used in the alignment are as follows; 1PD2_2_Rat_PGS – gi:6435744 (1PD2_2) from *Rattus norvegicus*; 1IYI_A_Homo_PGS – gi:30749302 (1IYI_A) from *Homo sapiens*; Ov_GST_Ia – gi:12005978 (AAG44695.1) from *Onchocerca volvulus*; Ov_GST_Ib – gi:12005978 (AAG44695.1) from *Onchocerca volvulus*; nOo.2.0.1.t09064– WormBase ParaSite (Armstrong *et al*., 2016). (B) This initial model produced *in silico* using SwissModel (Arnold *et al*., 2006) is based upon the alignment of the *O. ochengi* sequence with the Protein Databank template pdb.2HNL from the closely related *Onchocerca volvulus σ* GST. The dimeric protein model is shown here with the 25 disordered amino acids N-terminal extension. Blue, yellow and red are used to highlight the PDH_2_ binding pocket, predicted *N*-glycosylation sites and GSH binding sites respectively. Rotating the protein 90° shows the wide PDH_2_ binding pockets, revealing bound GSH (red ball and stick).

OoGST1 shares 99 and 96% identity to *O. volvulus* GST1b and 1a respectively (Fig. 1a). All three nematode GSTs are *σ* class, have a 25-amino acid signal peptide that is cleaved prior to maturation and possess a 25-amino acid N-terminal extension not found in any other GST to date. Sequence similarity to other *σ* class GSTs commences after this extension and, like OvGST1b, OoGST1 shows 32 and 35% identity to the human and rat haematopoietic prostaglandin D synthase (PGDS), respectively (accession numbers gi:30749302 and gi:6435744). Similarly, the proposed prostaglandin H_2_ binding pocket differs significantly between the mammalian and *Onchocerca* GSTs and may suggest a potentially different binding mode for OoGST1 and OvGST1b than for the rat and *Homo* PGDS (Perbandt *et al*., 2008).

There are also 6 potential *N*-glycosylation sites in OoGST1: one in the signal peptide (Asn^5^), one in the N-terminal extension (Asn^7^), two in the N-terminus (Asn^50^, Asn^79^), and two in the C-terminus (Asn^134^, Asn^144^) of the mature protein. Homology modelling of OoGST1 predicts, in accordance with other *σ* class GSTs, that this enzyme forms a dimeric protein (Fig. 1b). However, each homodimer possesses a ‘lock and key’ mechanism, typically observed only in other GST classes (*α*, *μ*, *π*) and vertebrate PGDS, but not normally observed in other nematodes aside from OvGST1b previously (Inoue *et al*., 2003; Line *et al*., 2019). Additionally, although topologically similar to *π* class GSTs, the structural differences in the substrate binding pocket of OoGST1 causes significant conformity changes resulting in a wider, shallower cleft.

The primary structure of the unusual 25 amino acid N-terminus extension is composed of a higher percentage (68%) of disorder-promoting amino acids; Ala, Arg, Gly, Gln, Ser, Glu, Lys and Pro, a low content of hydrophobic residues (12%), and no aromatic residues. This combination of amino acids suggests this part of the protein is unable to form the well-organized hydrophobic core that makes up a structured domain and thus is predicted to be an intrinsically disordered region (IDR) (Uversky, 2013). Indeed, x-ray crystallography of the OvGST1a on which this model was based revealed that this region lacked electron density and was therefore not modelled (Perbandt *et al*., 2008).

### Purification and enzymic characterization of OoGST

*Onchocerca ochengi* GST proteins from gravid, adult female worms were purified by affinity chromatography as described in Materials and Methods, and the enzymic activity carried out as shown by (Habig *et al*., 1974; Simons and Vander Jagt, 1977). Using CDNB as a model substrate, we found significant differences in cytosolic GST activity between whole worm extract, affinity-purified and column flow through (non-affinity) of 0.008, 1.310 and 0.001 *µ*mol min^−1^ mg^−1^, respectively (Supplementary Table 1). A 171-fold purification of GSTs was obtained, with a yield of almost 40% of the total GST activity content collected.

### Analysis of *O. ochengi* GSTs by 2DE

**Fig. 2.**
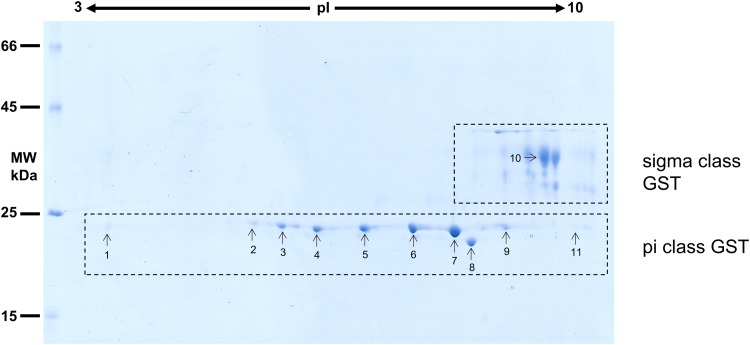
2DE analysis of cytosolic glutathione-binding proteins of *O. ochengi*. 20 *µ*g of *S*-HexylGSH purified GSTs were resolved *via* 2DE. Numbers/arrows indicate spots excised from the 2DE gel and identified *via* mass spectrometry (Table 1 and Supplementary file 1). Gel represents one of three ran independently with the same sample, with identified spots visualized in all three gels.

Considering that *O. ochengi* is predicted to have several GST classes (Armstrong *et al*., 2016), we carried out 2DE (Fig. 2) in order to resolve the GST classes for mass spectrometry protein identification (Table 1 and Supplementary file 1). Whilst *π* class GSTs are shown around the 25 kDa mark, but with different isoelectric points, *σ* class GSTs migrated at a higher apparent molecular mass (~35 kDa), but with spots less well-resolved than the *π* class isoforms. This indicates that *σ* class GST proteins were post-translationally modified, most likely glycosylated (see below).

**Table 1.** List of the most abundant proteins detected by mass spectrometry from in-gel analyses of *O. ochengi* GST

Spot Number	WormBase ID	Description	GST class	Score	% coverage	Predicted Mr (kDa)	Predicted PI	Peptide sequence
1	1::nOo.2.0.1.t00341	Glutathione transferase	*π*	70	24	24.4	7.3	R.LFLVDQDIK.F R.MIYMAYETEKDPYIK.S K.SQFQFGQLPCLYDGDQQIVQSGAILR.H
2	1::nOo.2.0.1.t00341	Glutathione transferase	*π*	49	19	24.4	7.3	K.LTYFSIR.G K.SILPGELAK.F R.LFLVDQDIK.F R.MIYMAYETEKDPYIK.S
3	1::nOo.2.0.1.t00341	Glutathione transferase	*π*	70	42	24.4	7.3	K.LTYFSIR.G K.SILPGELAK.F R.LFLVDQDIK.F R.MIYMAYETEKDPYIK.S R.KYNLNGENEMETTYIDMFCEGVR.D K.SQFQFGQLPCLYDGDQQIVQSGAILR.H
4	1::nOo.2.0.1.t00341	Glutathione transferase	*π*	91	42	24.4	7.3	K.LTYFSIR.G K.SILPGELAK.F R.LFLVDQDIK.F R.MIYMAYETEK.S R.MIYMAYETEKDPYIK.S R.KYNLNGENEMETTYIDMFCEGVR.D K.SQFQFGQLPCLYDGDQQIVQSGAILR.H
5	1::nOo.2.0.1.t00341	Glutathione transferase	*π*	150	42	24.4	7.3	K.LTYFSIR.G K.SILPGELAK.F R.LFLVDQDIK.F R.MIYMAYETEK.S R.MIYMAYETEKDPYIK.S K.YNLNGENEMETTYIDMFCEGVR.D R.KYNLNGENEMETTYIDMFCEGVR.D K.SQFQFGQLPCLYDGDQQIVQSGAILR.H
6	1::nOo.2.0.1.t00341	Glutathione transferase	*π*	259	42	24.4	7.3	K.LTYFSIR.G K.SILPGELAK.F R.LFLVDQDIK.F R.MIYMAYETEK.S R.MIYMAYETEKDPYIK.S K.YNLNGENEMETTYIDMFCEGVR.D K.SQFQFGQLPCLYDGDQQIVQSGAILR.H
7	1::nOo.2.0.1.t00341	Glutathione transferase	*π*	30	24	24.4	7.3	R.LFLVDQDIK.F R.MIYMAYETEKDPYIK.S K.SQFQFGQLPCLYDGDQQIVQSGAILR.H
8	1::nOo.2.0.1.t00341	Glutathione transferase	*π*	43		24.4	7.3	K.SILPGELAK.F R.LFLVDQDIK.F R.MIYMAYETEKDPYIK.S
9	1::nOo.2.0.1.t00341	Glutathione transferase	*π*	49	4	24.4	7.3	K.SILPGELAK.F R.LFLVDQDIK.F
10	1::nOo.2.0.1.t09064	Glutathione s-transferase 1	*σ*	231	34	28.5	9.4	K.IGQMPGIK.E K.LIPWTHEK.N K.YTLTYFNGR.N R.FGLLGTNAWEEAK.I K.LEEYPQLASFVNK.I K.IMAVVLNIEELFQK.L K.VSVADLAVFNMLMTLDDQVK.L
11	1::nOo.2.0.1.t00341	Glutathione transferase	*π*	109	19	24.4	7.3	K.LTYFSIR.G K.SILPGELAK.F R.LFLVDQDIK.F R.MIYMAYETEKDPYIK.S

Proteins were then excised from gels and identified by mass spectrometry in conjunction with MS-MS ions searches of a partially revised, publicly available *O. ochengi* database (Armstrong *et al*., 2016). Proteins identified were almost exclusively of the *π* and *σ* classes of GSTs (Table 1 and Supplementary file 1). As expected, we also identified bovine proteins, including a pi-class GST in several spots (Supplementary file 1).

### *Onchocerca ochengi* GSTs are *N*-glycosylated and mainly modified by oligomannose *N*-glycans

**Fig. 3.**
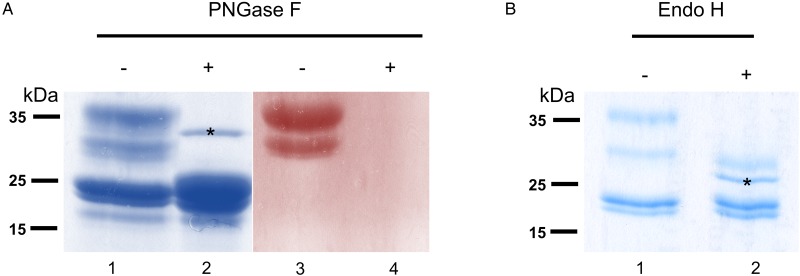
Glycosylated status of *S*-hexylGSH-purified *O. ochengi* GSTs. (A), 5 *µ*g of undigested (lanes 1 and 3) or PNGase F-treated (lanes 2 and 4) GSTs were fractionated on 12.5% SDS-PAGE and stained with either colloidal Coomassie blue (lanes 1 and 2) or PAS (lanes 3 and 4). The asterix (*) in lane 2 shows the migration of PNGase F enzyme. (B), Lanes 1 and 2 show non-glycosidase-digested and Endo H-treated GSTs from *O. ochengi* respectively. The asterix (*) in lane 2 shows Coomassie staining of the glycosidase Endo H enzyme.

To verify that *σ* class GSTs were glycosylated, as predicted by the presence of several *N*-glycosylation sequons (5–6) and also suggested by their migration on 2DE gels, *S-*hexylGSH-affinity-purified GSTs were incubated with either PNGase F or Endo-H. After digestion, only *σ* class GSTs appeared susceptible to either enzyme, as indicated by their faster migration on a Coomassie-stained gel (Fig. 3a and b). Furthermore, the lack of PAS reactivity after PNGase F treatment suggests that these proteins are specifically *N*-glycosylated (Fig. 3a, lanes 3 and 4). Whilst PNGase F caused a shift in migration of ~10 kDa, samples treated with Endo-H yield an extra band of ~28 kDa suggesting the possible presence of fucosylated, hybrid- or complex-type glycans on these proteins (Fig. 3b, lane 2). The identity of all GST proteins, before or after deglycosylation, was confirmed by mass spectrometry (not shown).

### *Onchocerca ochengi* GST are mainly modified by mannosylated *N*-glycans

**Fig. 4.**
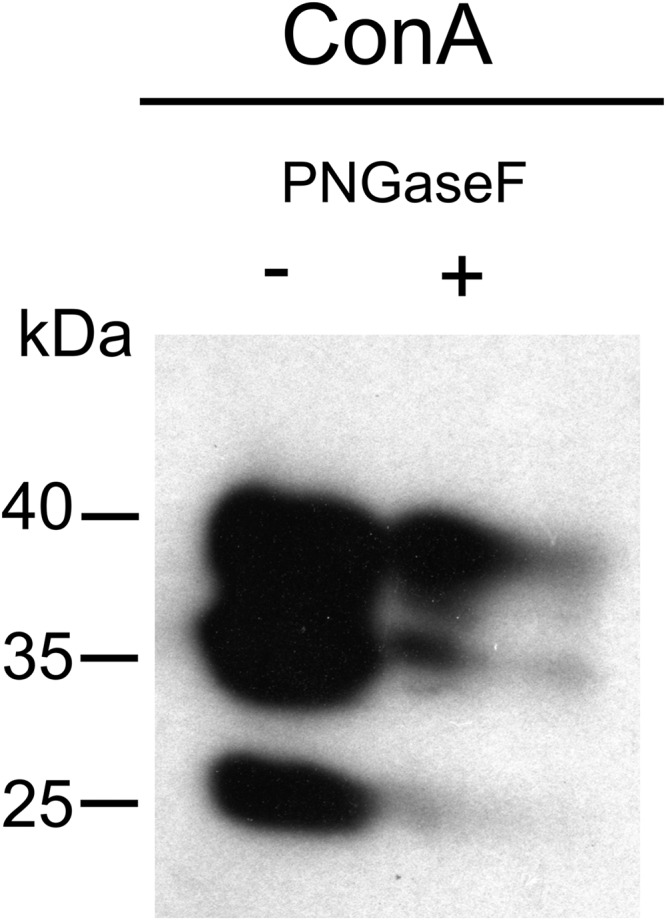
Lectin-affinity blotting of *O. ochengi* GST. Undigested (−) or PNGaseF-treated (+) GSTs were fractionated by SDS-PAGE, transferred to a nitrocellulose membrane and incubated with ConA for detection of mannose.

To further determine the types of *N*-glycans present on *O. ochengi* GST, lectin-blotting was carried out using ConA for the recognition of terminal *α*-mannose residues (Fig. 4). As expected, in the untreated sample, ConA recognized all the *σ* class GSTs, which migrated ~35–40 kDa, although recognition of a band with an apparent molecular mass of ~25 kDa suggests some degradation may have occurred (Fig. 4a). Following digestion with PNGaseF, most of the ConA binding was lost.

**Fig. 5.**
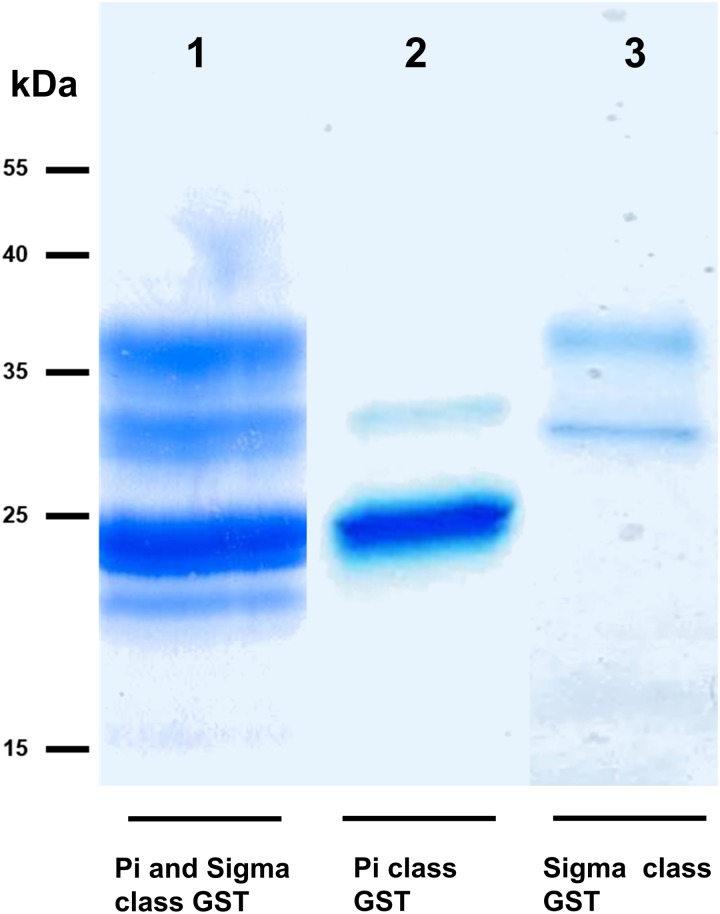
SDS PAGE gel showing *O. ochengi* glutathione transferases (GSTs), resolved *via* S-hexylGSH-affinity and ConA-lectin-affinity chromatography. All bands shown in the SDS PAGE image were glutathione transferases (GSTs) of *π* and *σ* classes, purified and identified *via* tandem mass spectrometry. Lane 1, GSTs of *π* and *σ* classes resolved from cytosolic extracts eluted from an *S*-hexylGSH-affinity column. Lane 2, *S*-hexylGSH-affinity GSTs of the *π* class that does not bind to the ConA-lectin-affinity column. Lane 3, ConA-lectin-binding *σ* class GSTs that also bind the *S*-hexylGSH-affinity column.

**Fig. 6.**
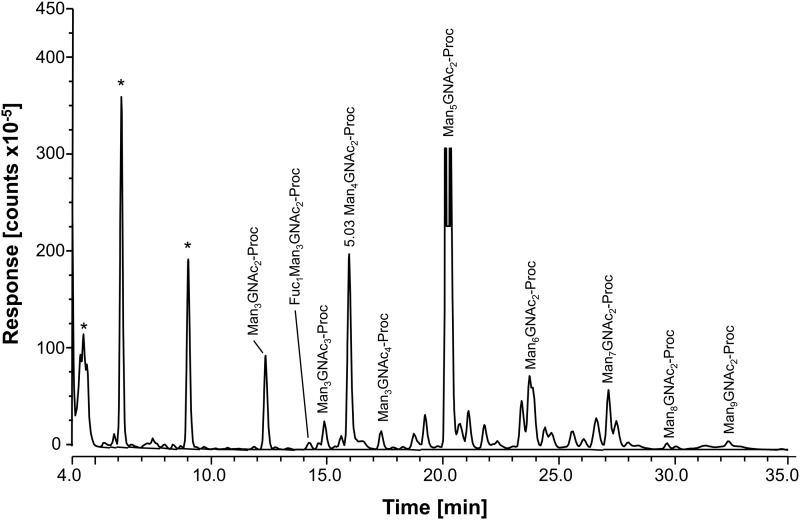
HILIC-LC separation of procainamide labelled *N*-glycans from *O. ochengi* GST1. Asterisks indicate contaminants (mainly from chitin hydrolysate ladder).

**Fig. 7.**
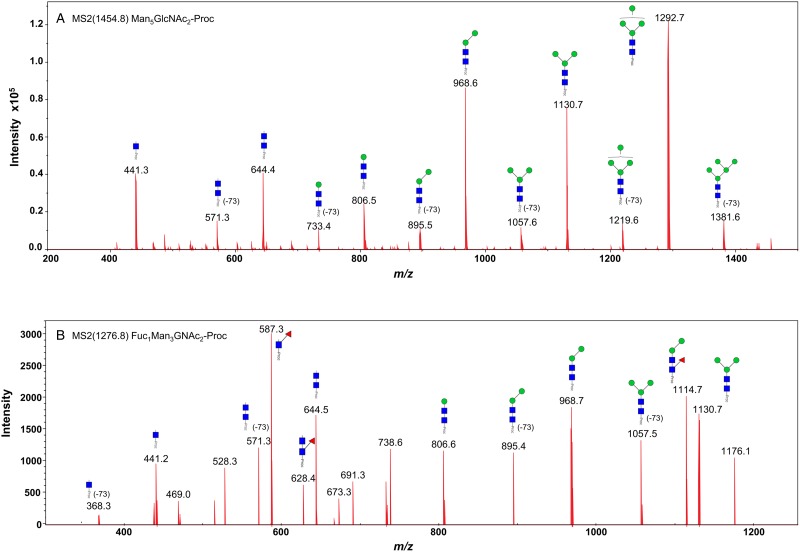
Positive-ion MS/MS spectra of Man_5_GlcNAc_2_-Pro (A) and Fuc_1_Man_3_GlcNAc_2_-Pro (B) from *O. ochengi* GST1. (−73) refers to [M + H^+^] ions that have lost terminal diethylamine from the procainamide tag during the collision (Kozak *et al*., 2015). Blue squares, *N*-acetylglucosamine residues; green circles, mannose residues; red triangles, fucose residues; Proc, procainamide tag.

We took advantage of ConA recognition of the *O. ochengi σ* class GSTs for protein purification and glycan structural analyses. After total *O. ochengi* GSTs were enriched by affinity chromatography using an *S*-hexylGSH column, only the glycosylated, *σ* class GSTs bound and were eluted off from the ConA column (Fig. 5). These samples were subsequently digested with PNGase F and the released glycans tagged with procanaimide and analysed by HILIC-liquid chromatography followed by ESI-MS and ESI-MS/MS. As shown in Fig. 6, the Man_5_GlcNAc_2_-Proc structure represents the main glycan species (~45%) from *σ* class OoGST. This was confirmed by positive-ion EIS-MS analysis, which showed the presence of abundant [M + H^+^] and [M + H^+^]^2+^ pseudomolecular ions at *m/z* 1454.8 and 727.9, respectively, corresponding to a glycan of composition Hex_5_HexNAc_2_-Proc (Fig. S1 and Table 2). In addition, short paucimmanose structures (Man_4_GlcNAc_2_-Proc and Man_3_GlcNAc_2_-Proc), oligomannoses (Man_6−9_GlcNAc_2_-Proc) and a few hybrid-type species (e.g. Fuc_1_Man_3_GlcNAc_2_-Proc and Man_3_GlcNAc_3−4_-Proc) were found (Table 2). The identity of all glycan species, including that of the Man_5_GlcNAc_2_-Proc oligosaccharide, was further corroborated by MS/MS analyses, which produced the characteristic fragment ions (Fig. 7a and b).

**Table 2.** Summary of most abundant *N*-glycan species from OoGST1

Peak No.	GU	% Relative abundance	Detected [M + Proc + 2H^+^]^2+^	Detected [M + Proc + H^+^]^1+^	Theoretical [M + Proc + H^+^]^1+^	Composition	Proposed structure
14	4.21	7.7	565.84	1130.67	1130.5	(Hex)_3_(HexNAc)_2_	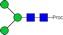
17	4.64	0.7	637.67	1276.69	1276.6	(Hex)_3_(HexNAc)_2_(Deoxyhexose)_1_	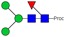
18	4.79	3.2	ND	1333.71	1333.3	(Hex)_3_(HexNAc)_3_	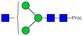
20	5.03	19.7	646.87	1292.73	1292.3	(Hex)_4_(HexNAc)_2_	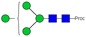
21	5.34	1.8	767.90	ND	1536.3	(Hex)_3_(HexNAc)_4_	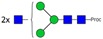
26	5.99	45.2	727.89	1454.78	1454.3	(Hex)_5_(HexNAc)_2_	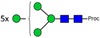
33	6.89	12.0	808.92	ND	1616.3	(Hex)_6_(HexNAc)_2_	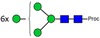
39	7.81	6.1	889.83	ND	1778.3	(Hex)_7_(HexNAc)_2_	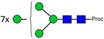
44	8.67	0.5	971.02	ND	1940.3	(Hex)_8_(HexNAc)_2_	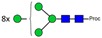
46	9.39	3.1	1054.55	ND	2102.3	(Hex)_9_(HexNAc)_2_	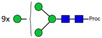

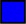
 GlcNAc; 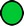 Mannose; 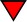 Fucose.

Proposed structures and relative abundance of the most common glycans from OoGST1 were taken from HILIC-LC (Fig. 6), and EIS-MS (Fig. S1) and EIS-MS/MS (Fig. 7) analyses.

### OoGST1 displays prostaglandin synthase activity

**Fig. 8.**
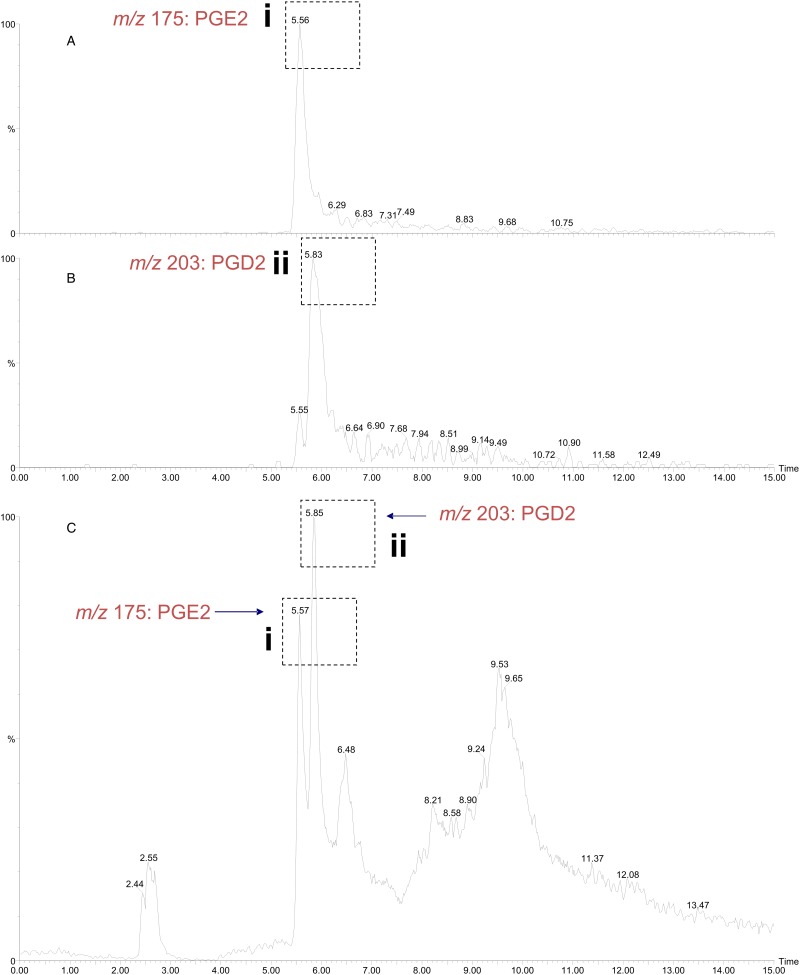
Detection of prostaglandin synthase activity of *O. ochengi σ* class GST *via* a mass spectrometry approach. A coupled assay with *O. ochengi* native *σ* class GST and COX-1 catalyses the conversion of arachidonic acid to the H2 form before the prostaglandin isomer is converted to either the D or E form. Nano-LC/MS analysis allowed detection of both PGE2 (A) and PGD2 (B) in the assay mixture with the PGD2 form being the more abundant of the two prostanoids (C). Dashed, boxed figures above peaks show the fragmentation ions specific to detection of PGE2 (i) and PGD2 (ii) according to the method used and described by LaCourse *et al*. (2012).

The *σ* class GSTs have previously been reported to synthesize prostaglandin D2 (PGD2), PGE2 and PGF2; eicosanoids that function in diverse physiological systems and pathological processes (Meyer and Thomas, 1995; Sommer *et al*., 2003). Using the ConA-affinity-purified *O. ochengi* GST fraction shown to contain only OoGST1 (Fig. 5, lane 3) we employed a coupled assay with cyclooxygenase (COX-1) and arachidonic acid which showed OoGST1 also has the ability to synthesize prostaglandins. Nano-LC/MS detected the presence of both PGD2 and PGE2 in the assay mixture with the PGD2 form being the more significantly abundant of the two eicosanoids (Fig. 8). OoGST1 appears to reflect a similar proportionality and specificity to catalyse predominantly, or only PGD2 from PGH2, in a concentration-dependent manner as described for OvGST1a (Sommer *et al*., 2003). The presence of PGE2, a relatively limited product observed, may, as proposed by Sommer *et al*. (2003) represent a by-product of rapid degradation of the highly unstable PGH2.

## Discussion

The *σ* class GST of *O. ochengi* is of particular interest in terms of the potential involvement in host immune modulation as well as possible roles in detoxification of other endogenous host- and parasite- derived toxins. Given the presence of a signal peptide and its detection at the host-parasite interface in bovine nodule fluid (Armstrong *et al*., 2016), suggesting probable roles in the long-term survival of the parasite, further investigation of this GST in *O. ochengi* is warranted. Furthermore, the ability of GSTs to detoxify endogenous and exogenous compounds, although well researched in other organisms, has not been fully explored in *O. ochengi*; that is, how any Phase II function may facilitate parasite establishment or survival.

We also show the predicted homology model between OvGST1a and OoGST1 is almost identical, with both homodimers forming a dimeric functional protein. The significance of the 25-amino acid disordered region that both GSTs possess is yet to be determined. Intrinsically disordered proteins (IDPs) and proteins containing IDRs lack stable tertiary and/or secondary structures under physiological conditions, but are nevertheless fully functional and actively participate in diverse functions mediated by proteins (Dunker *et al*., 2008; van der Lee *et al*., 2014). These functions include cell signalling, cell regulation, molecular recognition and have also been shown to modulate immune responses (Wright and Dyson, 2015). Recent evidence has shown that parasites such as *Schistosoma mansoni*, *Plasmodium falciparum* and *Toxoplasma gondii* overexpress several predicted disordered protein families, with transcripts more abundant in life stages that are exposed to the mammalian host immune system (Feng *et al*., 2006; Lopes *et al*., 2013; Ruy *et al*., 2014). Moreover, some of these families undergo disordered-to-ordered transitions in response to the external environmental conditions and have been shown to interact and bind with human immunomodulatory proteins (Lopes *et al*., 2013).

Although not tested here, the disordered region of OoGST1 may facilitate the way in which the GST is inserted at the host-parasite interface in the cuticular basal layer and the outer layer of the hypodermis. Interestingly, OoGST1 is the only *σ*-class *O. ochengi* GST that is expressed in all parasite life stages (Armstrong *et al*., 2016). Whilst several additional *σ*-class GSTs were previously detected in a shotgun proteomic analysis of the whole *O. ochengi* lifecycle, their absence in the current LC-MS study suggests they are expressed at a much lower level than OoGST1.

Here we have demonstrated that OoGST1 is able to synthesize two prostaglandins; PGD2 and PGE2. In line with studies on the *O. volvulus* OvGST1a (Sommer *et al*., 2003) PGD2 appears to predominate as the major isomerization product of the reaction. Research highlights the potential for prostaglandins to be involved in a variety of host-parasite interactions and roles including reproduction, inflammatory responses and immunomodulation, yet much still remains to be understood; specifically, how these mechanisms and roles may relate to GSTs in helminth parasites and their interactions with mammalian hosts (Daugschies and Joachim, 2000; Szkudlinski, 2000; Brattig *et al*., 2006; Kubata *et al*., 2007; Biserova *et al*., 2011; Joachim and Ruttkowski, 2011; Sankari *et al*., 2013; Kutyrev *et al*., 2017; Laan *et al*., 2017). Several reports highlight the potential involvement of eicosanoids in filarial worm infections. For example, Sommer *et al*. (2003) propose microfilariae of *O. vovlulus* present in the skin of humans may employ PGD2 to avoid the cutaneous immune response in a similar way to that demonstrated by Angeli *et al*. (2001) in the *Schistosoma mansoni*-mouse model of human infection. Lui and Weller (1992) demonstrate prostanoids secreted by *Brugia malayi* inhibited host platelet aggregation, whilst production of PGD2 in the supernatant of *Dirofilaria immitis* indicated involvement in the relaxation of the aorta during canine heartworm disease. Further studies exploring the potential of *Onchocerca*-derived PGD2 to modulate the host immune system are currently underway in our laboratory.

Our lectin-binding and structural analyses demonstrated that OoGST1 is a glycosylated protein. The expression of glycosylated GSTs has previously only been observed in the closely related filarial nematode, *O. volvulus*. OvGST1a and OvGST1b share 96 and 99% identity, respectively, to *O. ochengi* OoGST1, although the latter has an additional potential *N*-glycosylation sequon in the N-terminus. A combination of HILIC chromatography and LC-EIS-MS/MS of procainamide-labelled glycans showed that OoGST1 is mainly modified by a Man_5_GlcNAc_2_ oligosaccharide and, in a lower proportion, a series of larger oligomannose structures (i.e. Man_6−9_GlcNAc_2_). This is similar to the glycan profile observed in glycopeptides from OvGST1 (Sommer *et al*., 2001). Interestingly, we found that OoGST1 also contains a fucosylated hybrid-type structure, with the fucose residue potentially linked as *α*(1–3) to innermost GlcNAc residue (based on PNGase F sensitivity). Previous studies have shown that *N*-glycans containing an *α*(1–3)fucose residue are common in helminth glycoproteins, which are highly immunogenic and elicit TH2 immune responses (Faveeuw *et al*., 2003). It remains to be determined whether the presence of fucosylated glycans renders OoGST1 more immunogenic. Interestingly, OvGST1 *N*-glycans appear to render this protein more immunogenic to humans infected with *O. volvulus*, although no fucosylated glycans were detected by mass spectrometry (Sommer *et al*., 2001). Whilst the role of OoGST *N*-glycans remains to be determined, one potential function could be to increase its solubility in animal serum. Furthermore, the predominant presence of oligomannose structures could facilitate recognition by immune cell receptors with lectin domains, like the mannose receptor and DC-SIGN (Guo *et al*., 2004; Taylor *et al*., 2005).

Following this initial characterization of OoGST1, the opportunity exists for a wider range of studies upon this enzyme within the *O. ochengi* cattle experimental model system to inform studies of onchocerciasis and subsequent application to the human parasite *O. volvulus*. Indeed, cloning, expression and crystallographic studies, explorations of immunological aspects, as well as biochemical characterizations with a range of natural and model substrates, are underway with this glycosylated *σ* class GST of *O. ochengi*.

## Supplementary material

For supplementary material accompanying this paper visit https://doi.org/10.1017/S0031182019000763. click here to view supplementary material

## References

[ref1] Alhassan A, Makepeace BL, LaCourse EJ, Osei-Atweneboana MY and Carlow CK (2014) A simple isothermal DNA amplification method to screen black flies for *Onchocerca volvulus* infection. PLoS ONE 9, e108927. 25299656 10.1371/journal.pone.0108927 PMC4191976

[ref2] Angeli V, Faveeuw C, Roye O, Fontaine J, Teissier E, Capron A, Wolowczuk I, Capron M and Trottein F (2001) Role of the parasite-derived prostaglandin D2 in the inhibition of epidermal Langerhans cell migration during schistosomiasis infection. Journal of Experimental Medicine 193, 1135–1147. 11369785 10.1084/jem.193.10.1135 PMC2193325

[ref3] Armstrong SD, Xia D, Bah GS, Krishna R, Ngangyung HF, LaCourse EJ, McSorley HJ, Kengne-Ouafo JA, Chounna-Ndongmo PW, Wanji S, Enyong PA, Taylor DW, Blaxter ML, Wastling JM, Tanya VN and Makepeace BL (2016) Stage-specific proteomes from *Onchocerca ochengi*, sister species of the human river blindness parasite, uncover adaptations to a nodular lifestyle. Molecular & cellular proteomics: MCP 15, 2554–2575. 27226403 10.1074/mcp.M115.055640 PMC4974336

[ref4] Arnold K, Bordoli L, Kopp J and Schwede T (2006) The SWISS-MODEL workspace: a web-based environment for protein structure homology modelling. Bioinformatics (Oxford, England) 22, 195–201. 10.1093/bioinformatics/bti770 16301204

[ref5] Barrett J (1997) Biochemical pathways in parasites In Rogan MT (ed.), Analytical Parasitology. Berlin, Heidelberg: Springer Berlin Heidelberg, pp. 1–31.

[ref6] Biserova NM, Kutyrev IA and Malakhov VV (2011) The tapeworm *Diphyllobothrium dendriticum* (Cestoda) produces prostaglandin E2, a regulator of host immunity. Doklady Biological Sciences 441, 367–369. 22227682 10.1134/S0012496611060019

[ref7] Bradford MM (1976) A rapid and sensitive method for the quantitation of microgram quantities of protein utilizing the principle of protein-dye binding. Analytical Biochemistry 72, 248–254. 942051 10.1016/0003-2697(76)90527-3

[ref8] Brattig NW, Schwohl A, Rickert R and Buttner DW (2006) The filarial parasite *Onchocerca volvulus* generates the lipid mediator prostaglandin E(2). Microbes and Infection 8, 873–879. 16513382 10.1016/j.micinf.2005.10.014

[ref9] Brophy PM and Barrett J (1990) Glutathione transferase in helminths. Parasitology 100(Pt 2), 345–349. 2189115 10.1017/s0031182000061369

[ref11] Chasseaud LF (1979) The role of glutathione and glutathione S-transferases in the metabolism of chemical carcinogens and other electrophilic agents. Advances in Cancer Research 29, 175–274. 474272 10.1016/s0065-230x(08)60848-9

[ref12] Consortium IHG (2019) Comparative genomics of the major parasitic worms. Nature Genetics 51, 163–174. 30397333 10.1038/s41588-018-0262-1 PMC6349046

[ref13] Daugschies A and Joachim A (2000) Eicosanoids in parasites and parasitic infections. Advances in Parasitology 46, 181–240. 10761556 10.1016/s0065-308x(00)46009-4

[ref14] DeLano WL (2002) The PyMOL Molecular Graphics System. Available at http://www.pymol.org.

[ref15] Diemert DJ, Bottazzi ME, Plieskatt J, Hotez PJ and Bethony JM (2018) Lessons along the critical path: developing vaccines against human helminths. Trends in Parasitology 34, 747–758. 30064902 10.1016/j.pt.2018.07.005

[ref16] Dunker AK, Silman I, Uversky VN and Sussman JL (2008) Function and structure of inherently disordered proteins. Current Opinion in Structural Biology 18, 756–764. 18952168 10.1016/j.sbi.2008.10.002

[ref17] Faveeuw C, Mallevaey T, Paschinger K, Wilson IB, Fontaine J, Mollicone R, Oriol R, Altmann F, Lerouge P, Capron M and Trottein F (2003) Schistosome N-glycans containing core alpha 3-fucose and core beta 2-xylose epitopes are strong inducers of Th2 responses in mice. Eur J Immunol. 33, 1271–1281. 12731052 10.1002/eji.200323717

[ref18] Feng ZP, Zhang X, Han P, Arora N, Anders RF and Norton RS (2006) Abundance of intrinsically unstructured proteins in P. falciparum and other apicomplexan parasite proteomes. Molecular and Biochemical Parasitology 150, 256–267. 17010454 10.1016/j.molbiopara.2006.08.011

[ref19] Graham SP, Wu Y, Henkle-Duehrsen K, Lustigman S, Unnasch TR, Braun G, Williams SA, McCarthy J, Trees AJ and Bianco AE (1999) Patterns of *Onchocerca volvulus* recombinant antigen recognition in a bovine model of onchocerciasis. Parasitology 119(Pt 6), 603–612. 10633922 10.1017/s0031182099005065

[ref20] Guo Y, Feinberg H, Conroy E, Mitchell DA, Alvarez R, Blixt O, Taylor ME, Weis WI and Drickamer K (2004) Structural basis for distinct ligand-binding and targeting properties of the receptors DC-SIGN and DC-SIGNR. Nature Structural & Molecular Biology 11, 591–598. 10.1038/nsmb784 15195147

[ref21] Habig WH, Pabst MJ and Jakoby WB (1974) Glutathione S-transferases. The first enzymatic step in mercapturic acid formation. Journal of Biological Chemistry 249, 7130–7139. 4436300

[ref22] Hayes JD, Flanagan JU and Jowsey IR (2005) Glutathione transferases. Annual Review of Pharmacology and Toxicology 45, 51–88. 10.1146/annurev.pharmtox.45.120403.095857 15822171

[ref23] Howe KL, Bolt BJ, Shafie M, Kersey P and Berriman M (2017) Wormbase ParaSite – a comprehensive resource for helminth genomics. Molecular and Biochemical Parasitology 215, 2–10. 27899279 10.1016/j.molbiopara.2016.11.005 PMC5486357

[ref24] Inoue T, Irikura D, Okazaki N, Kinugasa S, Matsumura H, Uodome N, Yamamoto M, Kumasaka T, Miyano M, Kai Y and Urade Y (2003) Mechanism of metal activation of human hematopoietic prostaglandin D synthase. Natural Structural Biology 10, 291–296. 10.1038/nsb907 12627223

[ref25] Jakoby WB and Habig WH (1980) Glutathione transferases: In Jakoby WB (ed.), Enzymatic Basis of Detoxication. Academic Press: NY, London, Toronto, Sydney, San Francisco, II, pp. 63–94.

[ref26] Jaurigue JA and Seeberger PH (2017) Parasite carbohydrate vaccines. Frontiers in Cellular and Infection Microbiology 7, 248–248. 28660174 10.3389/fcimb.2017.00248 PMC5467010

[ref27] Joachim A and Ruttkowski B (2011) Prostaglandin D(2) synthesis in *Oesophagostomum dentatum* is mediated by cytosolic glutathione S-transferase. Experimental Parasitology 127, 604–606. 21056037 10.1016/j.exppara.2010.10.020

[ref28] Kallberg M, Wang H, Wang S, Peng J, Wang Z, Lu H and Xu J (2012) Template-based protein structure modeling using the RaptorX web server. Nature Protocols 7, 1511–1522. 22814390 10.1038/nprot.2012.085 PMC4730388

[ref29] Kelley LA, Mezulis S, Yates CM, Wass MN and Sternberg MJE (2015) The Phyre2 web portal for protein modeling, prediction and analysis. Nature Protocols 10, 845. 25950237 10.1038/nprot.2015.053 PMC5298202

[ref30] Kozak RP, Tortosa CB, Fernandes DL and Spencer DI (2015) Comparison of procainamide and 2-aminobenzamide labeling for profiling and identification of glycans by liquid chromatography with fluorescence detection coupled to electrospray ionization-mass spectrometry. Analytical Biochemistry 486, 38–40. 26079702 10.1016/j.ab.2015.06.006

[ref31] Kubata BK, Duszenko M, Martin KS and Urade Y (2007) Molecular basis for prostaglandin production in hosts and parasites. Trends in Parasitology 23, 325–331. 17531535 10.1016/j.pt.2007.05.005

[ref32] Kutyrev IA, Biserova NM, Olennikov DN, Korneva JV and Mazur OE (2017) Prostaglandins E2 and D2-regulators of host immunity in the model parasite *Diphyllobothrium dendriticum*: an immunocytochemical and biochemical study. Molecular and Biochemical Parasitology 212, 33–45. 28137629 10.1016/j.molbiopara.2017.01.006

[ref33] Laan LC, Williams AR, Stavenhagen K, Giera M, Kooij G, Vlasakov I, Kalay H, Kringel H, Nejsum P, Thamsborg SM, Wuhrer M, Dijkstra CD, Cummings RD and van Die I (2017) The whipworm (*Trichuris suis*) secretes prostaglandin E2 to suppress proinflammatory properties in human dendritic cells. FASEB Journal 31, 719–731. 27806992 10.1096/fj.201600841R PMC5240662

[ref34] LaCourse EJ, Hernandez-Viadel M, Jefferies JR, Svendsen C, Spurgeon DJ, Barrett J, Morgan AJ, Kille P and Brophy PM (2009) Glutathione transferase (GST) as a candidate molecular-based biomarker for soil toxin exposure in the earthworm *Lumbricus rubellus*. Environmental Pollution 157, 2459–2469. 19346039 10.1016/j.envpol.2009.03.015

[ref35] LaCourse EJ, Perally S, Morphew RM, Moxon JV, Prescott M, Dowling DJ, O'Neill SM, Kipar A, Hetzel U, E Hoey, Zafra R, Buffoni L, Perez Arevalo J and Brophy PM (2012) The Sigma class glutathione transferase from the liver fluke *Fasciola hepatica*. PLoS Neglected Tropical Diseases 6, e1666. 22666515 10.1371/journal.pntd.0001666 PMC3362645

[ref36] Laemmli UK (1970) Cleavage of structural proteins during the assembly of the head of bacteriophage T4. Nature 227, 680–685. 5432063 10.1038/227680a0

[ref37] Larkin MA, Blackshields G, Brown NP, Chenna R, McGettigan PA, McWilliam H, Valentin F, Wallace IM, A Wilm, Lopez R, Thompson JD, Gibson TJ and Higgins DG (2007) Clustal W and Clustal X version 2.0. Bioinformatics (Oxford, England) 23, 2947–2948. 10.1093/bioinformatics/btm404 17846036

[ref38] Liebau E, Wildenburg G, Walter RD and Henkle-Duhrsen K (1994) A novel type of glutathione S-transferase in *Onchocerca volvulus*. Infection and Immunity 62, 4762–4767. 7927752 10.1128/iai.62.11.4762-4767.1994 PMC303184

[ref39a] Liu LX and Weller PF (1992) Intravascular filarial parasites inhibit platelet aggregation. Role of parasite-derived prostanoids. J Clin Invest. 89, 1113–11120. 1313445 10.1172/JCI115691 PMC442967

[ref39] Line K, Isupov MN, LaCourse EJ, Cutress DJ, Morphew RM, Brophy PM and Littlechild JA (2019) X-ray structure of *Fasciola hepatica* Sigma class glutathione transferase 1 reveals a disulfide bond to support stability in gastro-intestinal environment. Scientific Reports 9, 902. 30696975 10.1038/s41598-018-37531-5 PMC6351632

[ref40] Lopes JLS, Orcia D, Araujo APU, DeMarco R and Wallace BA (2013) Folding factors and partners for the intrinsically disordered protein micro-exon gene 14 (MEG-14). Biophysical journal 104, 2512–2520. 23746524 10.1016/j.bpj.2013.03.063 PMC3672892

[ref41] Luk FC, Johnson TM and Beckers CJ (2008) N-linked glycosylation of proteins in the protozoan parasite *Toxoplasma gondii*. Molecular and Biochemical Parasitology 157, 169–178. 18096254 10.1016/j.molbiopara.2007.10.012 PMC2258246

[ref42] Makepeace BL and Tanya VN (2016) 25 years of the *Onchocerca ochengi* model. Trends in Parasitology 32, 966–978. 27665524 10.1016/j.pt.2016.08.013

[ref43] Meyer DJ and Thomas M (1995) Characterization of rat spleen prostaglandin H D-isomerase as a sigma-class GSH transferase. Biochemical Journal 311(Pt 3), 739–742. 7487927 10.1042/bj3110739 PMC1136065

[ref44] Meyer DJ, Muimo R, Thomas M, Coates D and Isaac RE (1996) Purification and characterization of prostaglandin-H E-isomerase, a sigma-class glutathione S-transferase, from *Ascaridia galli*. Biochemical Journal 313(Pt 1), 223–227. 8546687 10.1042/bj3130223 PMC1216886

[ref45] Perbandt M, Hoppner J, Burmeister C, Luersen K, Betzel C and Liebau E (2008) Structure of the extracellular glutathione S-transferase OvGST1 from the human pathogenic parasite *Onchocerca volvulus*. Journal of Molecular Biology 377, 501–511. 18258257 10.1016/j.jmb.2008.01.029

[ref46] Ruy PDC, Torrieri R, Toledo JS, Alves VDS, Cruz AK and Ruiz JC (2014) Intrinsically disordered proteins (IDPs) in trypanosomatids. BMC Genomics 15, 1100. 25496281 10.1186/1471-2164-15-1100 PMC4378006

[ref47] Sankari T, Hoti SL, Das LK, Govindaraj V and Das PK (2013) Effect of Diethylcarbamazine (DEC) on prostaglandin levels in *Wuchereria bancrofti* infected microfilaraemics. Parasitology Research 112, 2353–2359. 23525692 10.1007/s00436-013-3399-y

[ref48] Schmidt R, Coste O and Geisslinger G (2005) LC-MS/MS-analysis of prostaglandin E2 and D2 in microdialysis samples of rats. Journal of Chromatography. B. Analytical Technologies in the Biomedical and Life Sciences 826, 188–197. 16172029 10.1016/j.jchromb.2005.09.001

[ref49] Sheehan D, Meade G, Foley VM and Dowd CA (2001) Structure, function and evolution of glutathione transferases: implications for classification of non-mammalian members of an ancient enzyme superfamily. Biochemical Journal 360(Pt 1), 1–16. 11695986 10.1042/0264-6021:3600001 PMC1222196

[ref50] Simons PC and Vander Jagt DL (1977) Purification of glutathione S-transferases from human liver by glutathione-affinity chromatography. Analytical Biochemistry 82, 334–341. 907137 10.1016/0003-2697(77)90169-5

[ref51] Sommer A, Nimtz M, Conradt HS, Brattig N, Boettcher K, Fischer P, Walter RD and Liebau E (2001) Structural analysis and antibody response to the extracellular glutathione S-transferases from *Onchocerca volvulus*. Infection and Immunity 69, 7718–7728. 11705953 10.1128/IAI.69.12.7718-7728.2001 PMC98867

[ref52] Sommer A, Rickert R, Fischer P, Steinhart H, Walter RD and Liebau E (2003) A dominant role for extracellular glutathione transferase from *Onchocerca volvulus* is the production of prostaglandin D. Infection and Immunity 71, 3603–3606. 12761146 10.1128/IAI.71.6.3603-3606.2003 PMC155740

[ref53] Szkudlinski J (2000) Occurrence of prostaglandins and other eicosanoids in parasites and their role in host-parasite interaction. Wiadomosci Parazytologiczne 46, 439–446. 16886324

[ref54] Taylor PR, Martinez-Pomares L, Stacey M, Lin HH, Brown GD and Gordon S (2005) Macrophage receptors and immune recognition. Annual Review of Immunology 23, 901–944. 10.1146/annurev.immunol.23.021704.115816 15771589

[ref55] Thompson JD, Gibson TJ, Plewniak F, Jeanmougin F and Higgins DG (1997) The CLUSTAL_X windows interface: flexible strategies for multiple sequence alignment aided by quality analysis tools. Nucleic Acids Research 25, 4876–4882. 9396791 10.1093/nar/25.24.4876 PMC147148

[ref56] Trees AJ (1992) *Onchocerca ochengi*: Mimic, model or modulator of O. volvulus? Parasitology Today 8, 337–339. 15463530 10.1016/0169-4758(92)90068-d

[ref57] Trees AJ, Graham SP, Renz A, Bianco AE and Tanya V (2000) *Onchocerca ochengi* infections in cattle as a model for human onchocerciasis: recent developments. Parasitology 120(Suppl), S133–S142. 10874716 10.1017/s0031182099005788

[ref58] Uversky VN (2013) The alphabet of intrinsic disorder: II. Various roles of glutamic acid in ordered and intrinsically disordered proteins. Intrinsically Disordered Proteins 1, e24684–e24684. 28516010 10.4161/idp.24684 PMC5424795

[ref59] van der Lee R, Buljan M, Lang B, Weatheritt RJ, Daughdrill GW, Dunker AK, Fuxreiter M, Gough J, Gsponer J, Jones DT, Kim PM, Kriwacki RW, Oldfield CJ, Pappu RV, Tompa P, Uversky VN, Wright PE and Babu MM (2014) Classification of intrinsically disordered regions and proteins. Chemical Reviews 114, 6589–6631. 24773235 10.1021/cr400525m PMC4095912

[ref60] Vizcaino JA, Csordas A, del-Toro N, Dianes JA, Griss J, Lavidas I, Mayer G, Perez-Riverol Y, Reisinger F, Ternent T, Xu QW, Wang R and Hermjakob H (2016) 2016 update of the PRIDE database and its related tools. Nucleic Acids Research 44, D447–D456. 26527722 10.1093/nar/gkv1145 PMC4702828

[ref61] WHO (2018) Onchocerciasis Fact Sheet. Available at https://www.who.int/news-room/fact-sheets/detail/onchocerciasis.

[ref62] Wright PE and Dyson HJ (2015) Intrinsically disordered proteins in cellular signalling and regulation. Nature reviews. Molecular Cell Biology 16, 18–29. 25531225 10.1038/nrm3920 PMC4405151

[ref63] Xu D and Zhang Y (2011) Improving the physical realism and structural accuracy of protein models by a two-step atomic-level energy minimization. Biophysical Journal 101, 2525–2534. 22098752 10.1016/j.bpj.2011.10.024 PMC3218324

[ref64] Yang J, Yan R, Roy A, Xu D, Poisson J and Zhang Y (2015) The I-TASSER Suite: protein structure and function prediction. Nature Methods 12, 7–8. 25549265 10.1038/nmeth.3213 PMC4428668

