# Supplementary material for: Characterization of a novel glycosylated glutathione transferase of Onchocerca ochengi, closest relative of the human river blindness parasite
Source: Parasitology. 2019 Jul 3;146(14):1773–84. doi: 10.1017/S0031182019000763 (PMC6939172; doi:10.1017/S0031182019000763)
Supplement: Supplementary file 1 [file S0031182019000763sup.zip › S0031182019000763sup003.docx]

**Supplementary Table 1: Activity of purified *O. ochengi* GST.**

Mean values of each calculation in bold, and the standard deviation in italics. Calculations are based on the results of a standard Bradford assay and the glutathione activity assay.

| **MEAN VALUES (+/- SD) CONTROL (1, 2 & 3)** | **Total Protein (mg).** | **Total activity (Units) (µmol product per min.).**  *[Activity X Vol.]* | **Specific Activity (µmol.min-1.mg-1).** *[Activity ÷ Protein conc.]* | **Yield (%).**  *[Total activity ÷ Initial total activity X 100]* | **Fold purification.** *[Specific activity ÷ Initial specific activity]* |
| --- | --- | --- | --- | --- | --- |
| **Centrifuged, filtered homogenised sample.** | **37.845** *(16.240)* | **0.303** *(0.153)* | **0.008** *(0.001)* | **100** | **1** |
| **GSH affinity sample.** | **0.089** *(0.037)* | **0.113** *(0.042)* | **1.310** *(0.232)* | **39** *(6)* | **171** *(48)* |
| **Non-affinity sample.** | **27.309** *(10.949)* | **0.03** *(0.017)* | **0.001** *(0.001)* | **10** *(5)* | **0.14** *(0.07)* |
| **% GST yield soluble protein is 0.24,** *Std Dev +/- 0.05* | | | | | |
| **Theoretical total GST in worm (%) 0.63,** *Std Dev +/-* *0.16* | | | | | |
