# Supplementary figures and images for: Characterization of a novel glycosylated glutathione transferase of Onchocerca ochengi, closest relative of the human river blindness parasite
Source: Parasitology. 2019 Jul 3;146(14):1773–84. doi: 10.1017/S0031182019000763 (PMC6939172; doi:10.1017/S0031182019000763)

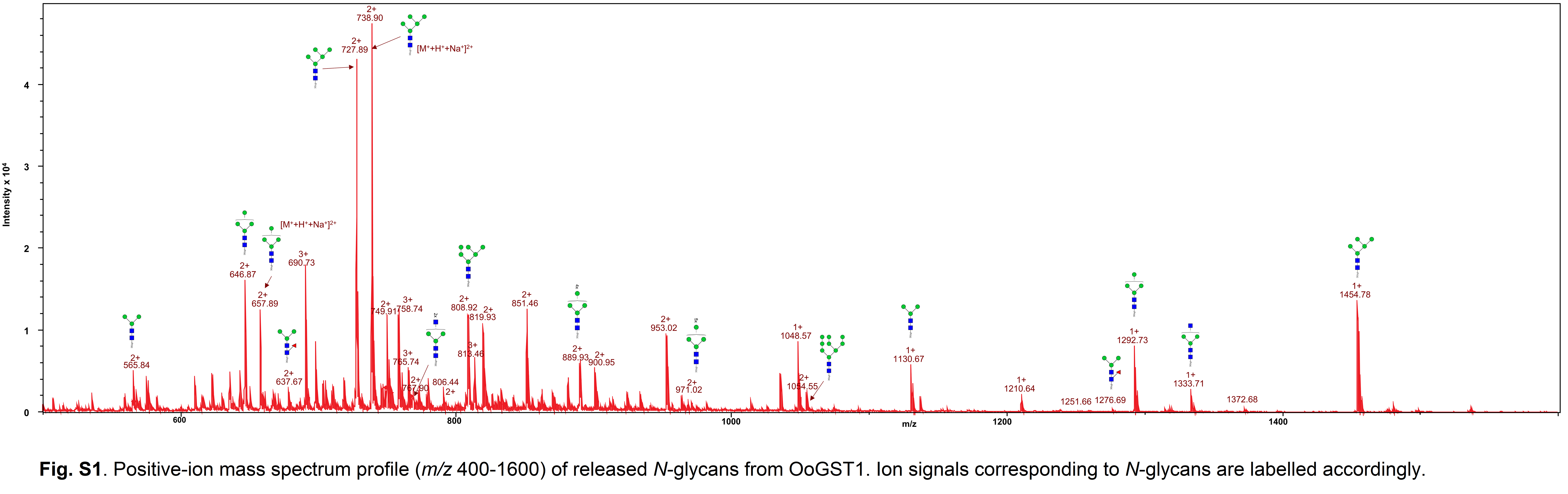

Supplement: Supplementary file 1 [file S0031182019000763sup.zip › S0031182019000763sup002.tif]
